# Changes in expression levels of *ERCC1, DPYD,* and *VEGFA* mRNA after first-line chemotherapy of metastatic colorectal cancer: results of a multicenter study

**DOI:** 10.18632/oncotarget.5227

**Published:** 2015-08-19

**Authors:** Hideo Baba, Yoshifumi Baba, Shinji Uemoto, Kazuhiro Yoshida, Akio Saiura, Masayuki Watanabe, Yoshihiko Maehara, Eiji Oki, Yasuharu Ikeda, Hiroyuki Matsuda, Masakazu Yamamoto, Mitsuo Shimada, Akinobu Taketomi, Michiaki Unno, Kenichi Sugihara, Yutaka Ogata, Susumu Eguchi, Seigo Kitano, Kazuo Shirouzu, Yasumitsu Saiki, Hiroshi Takamori, Masaki Mori, Toshihiko Hirata, Go Wakabayashi, Norihiro Kokudo

**Affiliations:** ^1^ Department of Gastroenterological Surgery, Graduate School of Medical Science, Kumamoto University, Kumamoto, Japan; ^2^ Department of Surgery, Graduate School of Medicine, Kyoto University, Kyoto, Japan; ^3^ Department of Surgical Oncology, Gifu Graduate School of Medicine, Gifu, Japan; ^4^ Department of Gastroenterological Surgery, Cancer Institute Hospital, Japanese Foundation for Cancer Research, Tokyo, Japan; ^5^ Department of Surgery and Science, Graduate School of Medical Sciences, Kyushu University, Fukuoka, Japan; ^6^ Department of Gastroenterological Surgery, National Hospital Organization Kyushu Cancer Center, Fukuoka, Japan; ^7^ Department of Surgery, Hiroshima Red Cross Hospital and Atomic-bomb Survivors Hospital, Hiroshima, Japan; ^8^ Department of Surgery, Tokyo Women's Medical University, Tokyo, Japan; ^9^ Department of Surgery, Institute of Health Biosciences, The University of Tokushima Graduate School, Tokushima, Japan; ^10^ Department of Gastroenterological Surgery I, Hokkaido University Graduate School of Medicine, Sapporo, Japan; ^11^ Department of Surgery, Tohoku University Graduate School of Medicine, Sendai, Japan; ^12^ Department of Surgical Oncology, Tokyo Medical and Dental University, Tokyo, Japan; ^13^ Department of Surgery, Kurume University Medical Center, Kurume, Japan; ^14^ Department of Surgery, Nagasaki University Graduate School of Biomedical Sciences, Nagasaki, Japan; ^15^ Department of Gastroenterological and Pediatric Surgery, Oita University Faculty of Medicine, Oita, Japan; ^16^ Department of Surgery, Kurume University School of Medicine, Kurume, Japan; ^17^ Coloproctology Center, Takano Hospital, Kumamoto, Japan; ^18^ Department of Surgery, Saiseikai Kumamoto Hospital, Kumamoto, Japan; ^19^ Department of Gastroenterological Surgery, Graduate School of Medicine, Osaka University, Osaka, Japan; ^20^ Department of Surgery, Japanese Red Cross Kumamoto Hospital, Kumamoto, Japan; ^21^ Department of Surgery, Iwate Medical University, School of Medicine, Morioka, Japan; ^22^ Hepato-Biliary-Pancreatic Surgery Division, Department of Surgery, Graduate School of Medicine, The University of Tokyo, Tokyo, Japan

**Keywords:** ERCC1, DPYD, VEGFA, colorectal cancer, bevacizumab

## Abstract

Our previous study showed that administering oxaliplatin as first-line chemotherapy increased ERCC1 and DPD levels in liver colorectal cancers (CRCs) metastases. Second, whether the anti-VEGF monoclonal antibody bevacizumab alters tumoral VEGFA levels is unknown. We conducted this multicenter observational study to validate our previous findings on ERCC1 and DPD, and clarify the response of VEGFA expression to bavacizumab administration. 346 CRC patients with liver metastases were enrolled at 22 Japanese institutes. Resected liver metastases were available for 175 patients previously treated with oxaliplatin-based chemotherapy (chemotherapy group) and 171 receiving no previous chemotherapy (non-chemotherapy group). *ERCC1*, *DPYD*, and *VEGFA* mRNA levels were measured by real-time RT-PCR. ERCC1 mRNA expression was significantly higher in the chemotherapy group than in the non-chemotherapy group (*P* = 0.033), and were significantly correlated (Spearman's correlation coefficient = 0.42; *P* < 0.0001). *VEGFA* expression level was higher in patients receiving bevacizumab (*n* = 51) than in those who did not (*n* = 251) (*P* = 0.007). This study confirmed that first-line oxaliplatin-based chemotherapy increases *ERCC1* and *DPYD* expression levels, potentially enhancing chemosensitivity to subsequent therapy. We also found that bevacizumab induces *VEGFA* expression in tumor cells, suggesting a biologic rationale for extending bevacizumab treatment beyond first progression.

## INTRODUCTION

Acquired resistance to chemotherapy and molecular-targeted therapy of human cancers is mediated by molecular alterations. Thus, understanding these alterations is increasingly important for predicting whether a patient will respond to chemotherapy and for counteracting resistance to anticancer agents.

The standard first-line chemotherapeutic regimen for metastatic colorectal cancer (CRC) is a combination of fluorouracil (5-FU) and folinic acid with oxaliplatin (i.e., FOLFOX) or irinotecan (i.e., FOLFIRI) with or without targeted agents.[1-3] Additionally, several second-line therapy regimes have been proposed for patients with recurring or progressive disease.[4-8] In a randomized phase II/III FIRIS study, IRIS (irinotecan/S-1) and FOLFIRI (5-FU–leucovorin/irinotecan) treatments yielded similar outcomes.[9, 10] Interestingly, this study reported a longer overall survival of patients in the IRIS group, previously undertaking oxaliplatin-containing chemotherapy, than patients in the FOLFIRI group. However, the reason for this remains poorly understood at the molecular level. To clarify the relevant molecular mechanisms, we previously conducted a single-center retrospective study of 45 CRC tissues. We found that administering oxaliplatin as first-line chemotherapy to CRC patients with liver metastases enhanced the patients' expression levels of two important genes: excision repair cross-complementing group 1 (*ERCC1*, a nucleotide excision repair pathway gene) and dihydropyrimidine dehydrogenase (*DPYD*, a pyrimidine catabolic pathway gene). We thus hypothesized that IRIS regimens combined with the DPD inhibitory fluoropyrimidine S-1 would be more effective against DPD-high tumors than the FOLFIRI regime.[11] However, our previous study was limited by a relatively small number of patients sourced from a single institute.

Bevacizumab is an anti-vascular endothelial growth factor (VEGF) monoclonal antibody commonly included in first-line therapy of metastatic CRC.[12, 13] Once disease has progressed beyond first line chemotherapy, maintaining VEGF inhibition by bevacizumab has proven a clinically beneficial adjunct to standard second-line chemotherapy.[14-18] However, the biological rationale of continuing bevacizumab beyond first progression remains elusive. Given that circulating levels of short vascular endothelial growth factor A (VEGFA) isoforms and genetic variants of *VEGFA* or its receptors are promising biomarker candidates for bevacizumab,[19] we propose that investigating the *VEGFA* expression levels before and after first-line bevacizumab treatment may help to elucidate this rationale.

The present multicenter observational study of 346 CRC patients validates our previous findings that *ERCC1* and *DPYD* expression levels are altered by oxaliplatin-based chemotherapy. We also evaluate the response of *VEGFA* expression levels to bevacizumab administration.

## RESULTS

### Patient characteristics

Table 1 summarizes the characteristics of the study patients. The mean patient age at the time of liver dissection was 64.5 years (range 32–89 years). Oxaliplatin as first-line chemotherapy was administered to 166 patients under the following regimes: FOLFOX (92 patients), FOLFOX + Bevacizumab (52 patients), XELOX+ Bevacizumab (5 patients), XELOX (3 patients), and other regimens (14 patients).

**Table 1:** Patients characteristics

Clinical or pathological feature	Total N	Prior oxaliplatin-based chemotherapy		*P* value
		No	Yes	
All cases	336	170	166	
Mean age ± SD	64.5 ± 10.7	66.3 ± 10.4	62.7 ± 10.7	0.0019
Sex				0.23
Male	217 (65%)	115 (68%)	102 (61%)	
Female	119 (35%)	55 (32%)	64 (39%)	
Number of liver metastasis				0.0002
1	146 (43%)	91 (53%)	55 (33%)	
2-	190 (57%)	79 (47%)	111 (67%)	
Tumor location				0.18
Proximal colon	70 (21%)	41 (24%)	29 (18%)	
Distal colon	135 (40%)	70 (41%)	65 (39%)	
Rectum	131 (39%)	59 (35%)	72 (43%)	
Tumor differentiation				0.12
Well	95(28%)	56 (33%)	39 (24%)	
Moderate	219 (65%)	102 (60%)	117 (70%)	
Others	22 (6.6%)	12 (7.1%)	10 (6.0%)	
Prior chemotherapy				
None		170 (100%)		
mFOLFOX6			92 (55%)	
mFOLFOX+bevacizumab			52 (33%)	
XELOX+bevacizumab			5 (3.0%)	
XELOX			3 (1.8%)	
Others			14 (8.4%)	

(%) indicates the proportion of cases with a specific clinical or pathological feature among each group (prior chemotherapy Yes or No)

Patients in the oxaliplatin-based chemotherapy group (chemotherapy group) were generally younger (P = 0.0014) and had more liver metastases (*P* = 0.0001) than patients in the non-chemotherapy group (Table 1). The mean number of liver metastases in the chemotherapy group was 3.51 (range 1–19), versus 2.14 in the non-chemotherapy group (range 1–14) (*P* < 0.0001). There was no significant difference in sex, tumor location, or tumor differentiation between the two groups.

### *ERCC1* and *DPYD* mRNA expression levels with and without a prior oxaliplatin regimen

As shown in Figure 2, *ERCC1* mRNA expression was significantly higher in the chemotherapy group (mean 7.11; median 7.12) than in the non-chemotherapy group (mean 6.94; median 6.88) (*P* = 0.033). *DPYD* mRNA expression was similarly elevated in the chemotherapy group (mean 5.32; median 5.17) relative to the non-chemotherapy group (mean 5.04; median 5.17) (*P* = 0.023). In the chemotherapy group, ERCC1 or *DPYD* mRNA levels were unassociated with the number of chemotherapeutic cycles and with type of chemotherapeutic regimen (data not shown). However, expression levels of *ERCC1* and *DPYD* were significantly correlated (Spearman's correlation coefficient = 0.42; *P* < 0.0001) (Figure 3), consistent with the findings of our previous single-center study.[11]

**Figure 1:**
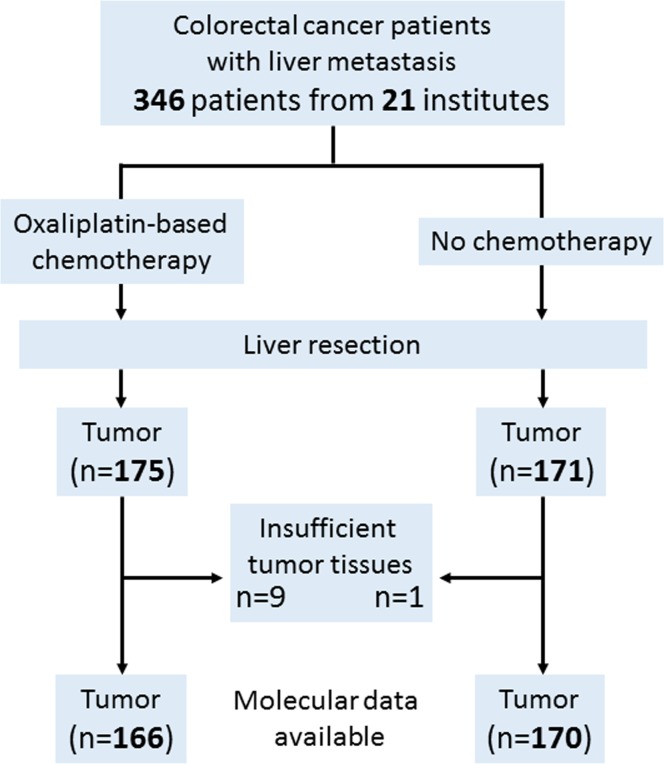
Flowchart of the present study

**Figure 2:**
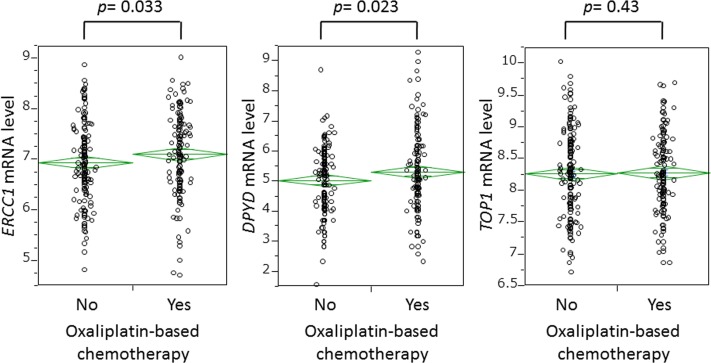
Comparison of expression levels of *ERCC1*, *DPYD*, and *TOP1* genes in tumor cells with and without oxaliplatin-based chemotherapy before hepatectomy

**Figure 3:**
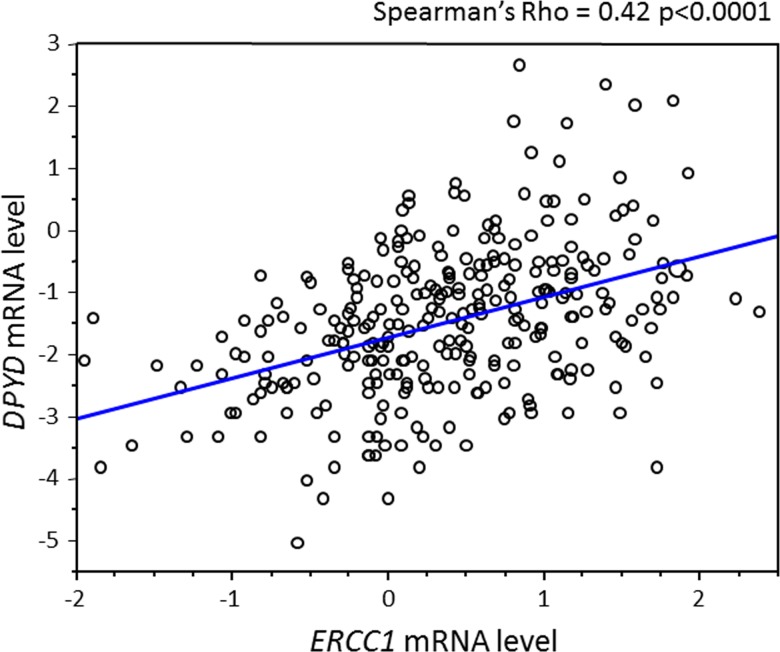
Relationship between *ERCC1* and *DPYD* expression levels

Given that chemotherapy history was significantly related to patient age and number of liver metastasis (Table 1), we correlated mRNA levels of *ERCC1* and *DPYD* with both parameters. Age was not associated with *ERCC1* (Spearman's correlation coefficient = −0.02; *P* = 0.51) or *DPYD* mRNA level (Spearman's correlation coefficient = −0.04; *P* = 0.71). Similarly, no relationship was found between number of liver metastasis and *ERCC1* or *DPYD* mRNA levels (*P* = 0.69 and *P* = 0.76 for *ERCC1* and *DPYD*, respectively).

We also examined whether a prior oxaliplatin regimen altered the mRNA expression of DNA topoisomerase I (*TOP1*), a recognized predictive biomarker of irinotecan therapy. No significant difference in *TOP1* mRNA level was found between the groups receiving and not receiving oxaliplatin (Figure 2).

### Immunohistochemical results

The RT–PCR analysis revealed higher expression of *ERCC1* and *DPYD* mRNA in oxaliplatin-treated patients than in non-treated patients. The protein expression levels of these genes were determined by immunohistochemical examination. Tumor cells contained appreciable quantities of ERCC1 protein, especially in the nucleus, whereas both tumor and stromal cells expressed DPD protein (Supplemental figure 1). One of the investigators, blinded to all other participant data, classified nuclear ERCC1 and DPD expression as absent, weak, moderate, or strong. Tumors with weak to strong expression were defined as “positive,” and tumors not expressing these proteins were defined as “negative.” Among 340 colorectal liver metastases, 181 (55%) and 131 (39%) tumors tested positive for ERCC1 and DPD, respectively. Importantly, ERCC1 and DPD positivity was significantly associated with mRNA expression levels for each gene (*P*=0.0042 for DPD, P<0.001 for ERCC1). Positive DPD was significantly associated with prior oxaliplatin-based chemotherapy (*p* = 0.027; see Supplemental Table 2), consistent with the RT–PCR results. Conversely, ERCC1 expression was unrelated to prior oxaliplatin-based chemotherapy (*p* = 0.44; Supplemental Table 2).

### *VEGFA* expression level and bevacizumab

**Figure 4:**
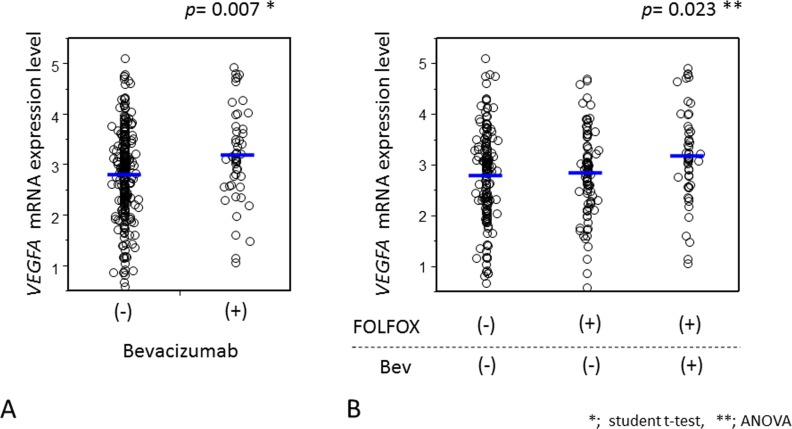
Comparison of *VEGFA* expression levels in tumor cells with and without bevacizumab treatment before hepatectomy

To test the effect of bevacizumab on *VEGFA* mRNA expression, we evaluated the mRNA expression level of *VEGFA* in the presence and absence of bevacizumab therapy. In this study, 63 patients had received a prior bevacizumab-including regimen, while 277 patients had not received this therapy. Among the non-bevacizumab group, 172 and 105 patients had received a prior oxaliplatin-including regimen and no chemotherapy, respectively. Results of tumoral *VEGFA* mRNA were available in 301 patients. Importantly, *VEGFA* mRNA expression level was higher in patients receiving bevacizumab (n = 51; mean = 3.18, median = 3.12) than in their non-bevacizumab counterparts (n = 251; mean = 2.81, median = 2.89) (*p* = 0.007) (Figure 4A). Additionally, we found that whereas *VEGFA* mRNA expression levels were unaffected by oxaliplatin-based chemotherapy, they were significantly altered by bevacizumab (*P* = 0.014) (Figure 4B). The bevacizumab-including regimen did not influence the expression levels of *ERCC1*, *DPYD*, or TOP1 (*P* > 0.10).

## DISCUSSION

Our multicenter study of 346 CRC patients revealed significantly higher *ERCC1* and *DPYD* expression in patients receiving oxaliplatin as a first-line chemotherapy than in patients receiving no chemotherapy. Given that IRIS (irinotecan/S-1) regimens based on the DPD inhibitor fluoropyrimidine may be more effective against DPD-high tumors than FOLFIRI, this finding is consistent with a recent clinical study, which suggested that patients previously treated with oxaliplatin-based chemotherapy better responded to IRIS than to FOLFIRI.[9, 10] Second, we found that administering bevacizumab to patients raised their *VEGFA* expression levels, supporting that bevacizumab encourages *VEGFA* mRNA expression from tumor cells via feedback or alternative unknown mechanisms. This phenomenon provides a possible biologic rationale for continuing bevacizumab after first progression.

Molecular responses to chemotherapy and molecular-targeted therapy have been implicated in acquired resistance to these therapies. Thus, by better understanding these molecular alterations, we may select a more effectual second-line regimen. To our knowledge, we present the first demonstration of a basic rationale for second-line therapy following failure of oxaliplatin-based first-line therapy in CRC patients. Additionally, we hypothesized the biologic rationale for continuing bevacizumab treatment after first progression. In this context, the clinical implications of our study could be considerable.

**Figure 5:**
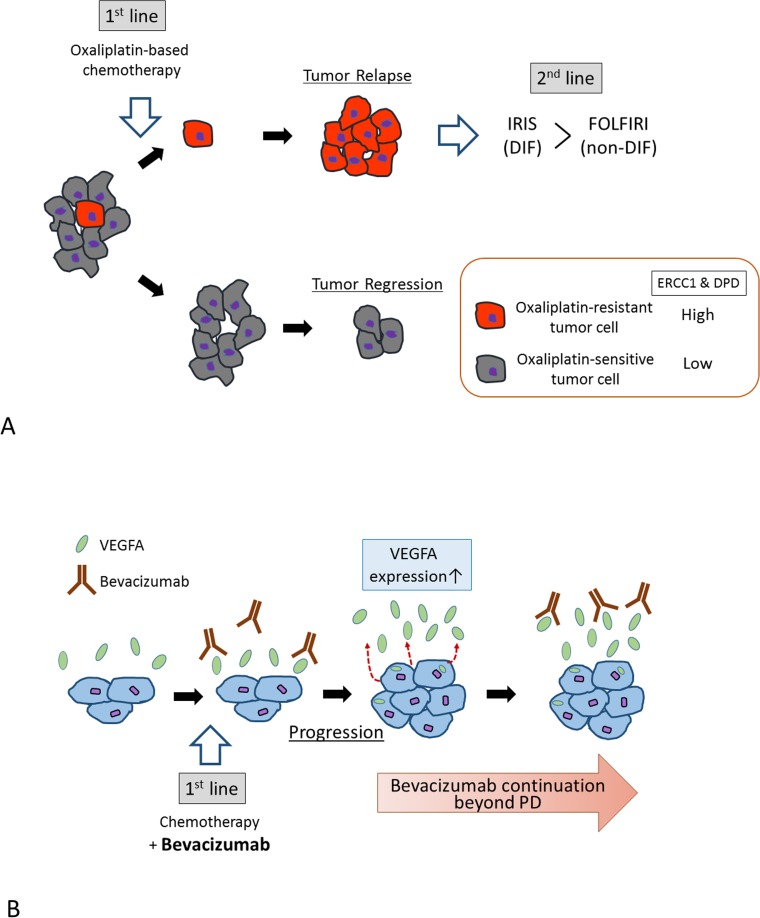
**A.** Proposed molecular mechanism underlying the superiority of IRIS treatment in prior oxaliplatin-treated patients. Oxaliplatin-resistant tumor cells may be sensitized to IRIS therapy by their high ERCC1 and DPD levels. **B.** Proposed molecular mechanism rationalizing continued bevacizumab treatment after first progression. Bevacizumab may encourage *VEGFA* mRNA expression from tumor cells via an unknown feedback mechanism.

Although a previous analysis found that IRIS was superior to FOLFIRI in patients previously treated with oxaliplatin-based chemotherapy,[9] the molecular mechanisms underlying this finding were not clarified. In our previous *in-silico* study of cell-line panel data retrieved from the National Cancer Institute 60 (NCI60), oxaliplatin and 5-FU sensitivities were significantly correlated, and cells resistant to oxaliplatin showed significantly higher ERCC1 and DPD expression than sensitive cells.[11] Clinical samples also confirmed that the cancer cells of FOLFOX-treated patients expressed significantly more ERCC1 and DPD than cells of non-treated patients. Based on these findings, we propose the following hypothesis (Figure 5A). Following first-line oxaliplatin-based treatment, oxaliplatin-sensitive tumor cells (with low ERCC1 levels; colored gray in Figure 5A) are killed, whereas a small fraction of relatively oxaliplatin-resistant cells (with high ERCC1 levels; colored red in Figure 5A) survive. The NCI60 cell-line data reveal *ERCC1* and *DPYD* gene expressions as confounding factors; therefore, surviving cells will express high levels of both *ERCC1* and *DPYD*. As the IRIS regimen contains the DPD inhibitory fluoropyrimidine S-1,[20] it will more effectively target DPD-high tumors than FOLFIRI (based on fluoropyrimidine, which does not inhibit DPD). This hypothesis was supported in the current study of more than 300 CRC samples retrieved from multiple centers. Of course, these findings must be consolidated by further studies. We also need to elucidate the molecular mechanisms underlying the confounding effects of *ERCC1* and *DPYD* gene expression in cancer cells.

Continuing VEGF inhibition by bevacizumab treatment beyond disease progression is widely accepted as beneficial for patients with metastatic CRC. [14-18] According to the “normalization” hypothesis, bevacizumab instigates a redistribution of tumor blood flow, increasing the delivery of chemotherapy to the tumor.[21, 22] Another possible mechanism is treatment-related changes in VEGFA, although attempts to predict the effect of bevacizumab on tumor or plasma VEGFA levels have been largely inconsistent. Several clinical studies have demonstrated that bevacizumab delivery elevates levels of circulating VEGFA.[19, 23, 24] Nonetheless, to our knowledge, the effect of bevacizumab on tumoral VEGFA levels has not been previously reported. CRC cells exposed to bevacizumab increase their *VEGFA* gene expression, with consequent increases in tumor cell migration and invasion, and metastatic potential *in vivo*.[25] Collectively, our findings suggest that bevacizumab encourages *VEGFA* mRNA expression in tumor cells via an unknown feedback mechanism. Therefore, bevacizumab is a clinical necessity even after first progression (Figure 5B).

There are advantages in accessing the databases of multiple centers. The importance of large-scale studies cannot be overemphasized, because small studies yielding significant results are much more likely to be published than those yielding null results, leading to publication bias. The 346 participants in the present study were treated at 22 hospitals throughout Japan. Thus, this sample better represents the Japanese CRC population than samples collected from a few academic hospitals. The limitations of a multiple database study are that resected specimen-handling procedures (such as taking samples and preparing formalin-fixed paraffin-embedded tissues) subtly differ among medical centers. This may explain our unexpected result that ERCC1 immunostaining is independent of prior oxaliplatin-based chemotherapy. Another limitation may be the lack of tumor specimens which are obtained before chemotherapeutic treatment. The current retrospective study could not examine the relationship between pre-treatment status of *ERCC1* and *DPYD* expression and therapeutic response. In addition, we could not set the cut-offs for these molecular markers toward the future practical application in the clinical setting. We acknowledge that a well-planned prospective study is necessary to overcome these limitations.

In summary, this multicenter study revealed that *ERCC1* and *DPYD* expression levels are increased by first-line oxaliplatin-based chemotherapy, with possible impacts on chemosensitivity to subsequent therapy. Second, we found that bevacizumab administration boosts *VEGFA* expression levels in the tumors of receiving patients, providing a possible biologic rationale for continuing bevacizumab treatment after first progression.

## MATERIALS AND METHODS

### Study subjects

This retrospective study included 346 CRC patients who underwent hepatectomy for liver metastasis between April 2005 and October 2013 at 22 institutions in Japan. All tumors were histologically diagnosed as adenocarcinomas of the colon or rectum. As the aim of this study was to compare the expression levels of *ERCC1*, *DPYD* and *VEGFA* between patients receiving oxaliplatin-based chemotherapy and patients receiving no chemotherapy, we enrolled both types of patients equally. As a result, 171 patients had undergone no chemotherapy prior to hepatectomy, and 175 patients had received oxaliplatin-based chemotherapy [5-fluorouracil, leucovorin, and oxaliplatin (FOLFOX) (92 cases), FOLFOX + Bevacizumab (58 cases), capecitabine and oxaliplatin (XELOX) (5 cases), XELOX + Bevacizumab (5 cases), other treatments (15 cases)] prior to hepatectomy. On average, the chemotherapy group had received 8 courses (cycles) of treatment (range 2–34 courses). We initially subjected 346 cancer specimens to molecular analyses (i.e., immunohistochemical staining and quantitative reverse transcription polymerase chain reaction (RT-PCR), and retrieved valid results from 336 (97%) of the specimens. Thus, 336 colorectal tumors (166 from the oxaliplatin-based chemotherapy group and 170 from the no-chemotherapy group) were finally included in this study (Figure 1). The study protocol was approved by the independent ethics committee of each participating institution.

### Quantitative RT-PCR

The protocols of RNA isolation and cDNA synthesis are shown in Supplemental data 1. Gene expression levels of *ERCC1*, *DPYD*, topoisomerase-1 (*TOP1*) and *VEGFA* were determined by TaqMan real-time PCR (Life Technologies, Foster City, CA) as previously described.[26, 27] The endogenous reference gene was β-Actin (*ACTB*). All genes from all samples were run in triplicate. The smallest detectable quantity of amplified cDNA defines the cycle threshold (Ct) value, which is inversely proportional to the cDNA content. Universal Mix RNAs (Stratagene, La Jolla, CA) were used as control calibrators on each plate. The primer sequences for *ERCC1, DPYD, TOP1, VEGFA* and *ACTB* were as previously described. [27, 28] The adopted primers and probes are listed in Supplemental Table 1.

The PCR reaction mixture comprised 1,200 nmol/L of each primer, 200 nmol/L probe, 0.4 units of AmpliTaq Gold Polymerase, 200 nmol/L each of dATP, dCTP, dGTP, dTTP, 3.5 mmol/L MgCl2, and 1× Taqman buffer A containing a reference dye. Reagents (all purchased from Life technologies, Foster City, CA) were combined in a final volume of 20 μL. Cycling conditions were 50°C for 2 minutes and 95°C for 10 minutes, followed by 46 15-second cycles at 95°C and 60°C for 1 minute. The threshold cycle (Ct) was the fractional cycle number at which the fluorescence generated by the probe cleavage exceeded a fixed level above baseline. The relative amount of tissue target mRNA, standardized against the amount of ACTB mRNA, was expressed as −ΔCt = − (Ct(target gene-1) − Ct(β-actin)). The number ratio of target mRNA copies to ACTB mRNA copies was then calculated as 2−ΔCt × K, where K is a constant.[29] To prevent significant contamination by genomic DNA, we amplified non-reverse-transcribed RNA only.

### Immunohistochemical staining

In preparation for ERCC1 and DPD analyses, the slides were incubated overnight at 4°C with primary anti-ERCC1 monoclonal antibody (Clone D-10; Santa Cruz Biotechnology, Inc., Santa Cruz, CA) and primary anti-DPD monoclonal antibody (Clone OF-303, Taiho Pharmaceutical Co., Ltd, Tokyo, Japan), respectively. Both antibodies were diluted by a factor of 100. The secondary antibody was a ready-to-use anti-mouse EnVision-Peroxidase system (Dako Japan Inc., Tokyo, Japan). The remaining procedure was performed using a Dako EnVision+ System (Dako Japan Inc). The chromogenic detection substrate was DAB (3,30-diaminobenzidine). The stained slides were counterstained with hematoxylin and bluing reagent.

### Statistical methods

Categorical data were analyzed by the w2 test. Inter-group differences were evaluated by Student's t-test or the Wilcoxon test. Results were considered statistically significant at the P < 0.05 level. All statistical analyses were performed by JMP version 8.01 software (SAS Institute Inc., Cary, NC).

## SUPPLEMENTARY MATERIAL TABLES AND FIGURE


