# Erratum: Changes in expression levels of *ERCC1*, *DPYD*, and *VEGFA* mRNA after first-line chemotherapy of metastatic colorectal cancer: results of a multicenter study

**Published:** 2015-11-16

**Authors:** Hideo Baba, Yoshifumi Baba, Shinji Uemoto, Kazuhiro Yoshida, Akio Saiura, Masayuki Watanabe, Yoshihiko Maehara, Eiji Oki, Yasuharu Ikeda, Hiroyuki Matsuda, Masakazu Yamamoto, Mitsuo Shimada, Akinobu Taketomi, Michiaki Unno, Kenichi Sugihara, Yutaka Ogata, Susumu Eguchi, Seigo Kitano, Kazuo Shirouzu, Yasumitsu Saiki, Hiroshi Takamori, Masaki Mori, Toshihiko Hirata, Go Wakabayashi, Norihiro Kokudo

Oncotarget. 2015; 6:34004-34013

***PMID***: *26372896*

Present:

**ACKNOWLEDGMENTS**

Our study was supported by unrestricted technical assistance from Taiho Pharmaceutical Co., Ltd, Japan. We thank Kazuto Harada, Keisuke Kosumi, and Keisuke Miyake for their technical support. We also thank Takashi Kobunai for his helpful advice.

Corrected:

**ACKNOWLEDGMENTS**

Financial support for this research was provided in part by Taiho Pharmaceutical Co., Ltd, Japan. We thank Kazuto Harada, Keisuke Kosumi, and Keisuke Miyake for their technical support. We also thank Takashi Kobunai for his helpful advice.

