# Supplementary material for: Changes in expression levels of ERCC1, DPYD, and VEGFA mRNA after first-line chemotherapy of metastatic colorectal cancer: results of a multicenter study
Source: Oncotarget. 2015 Aug 19;6(32):34004–13. doi: 10.18632/oncotarget.5227 (PMC4741821; doi:10.18632/oncotarget.5227)
Supplement: Supplementary file 1 [file oncotarget-06-34004-s001.pdf]

Changes in expression levels of *ERCC1, DPYD*, and *VEGFA* mRNA after first-line chemotherapy of metastatic colorectal cancer: results of a multicenter study

Supplementary Material

Supplemental table 1. Primer and probe sequences for quantitative RT–PCR

Gene	Forward primer (5'-3')	Reverse primer (5'-3')	Taqman® probe (5'-3')	Amplicon size(bp)
ERCC1	GGGAATTTGGCGACGTAATTC	GCGGAGGCTGAGGAACAG	CACAGGTGCTCTGGCCCAGCACATA	71
DPD	AGGACGCAAGGAGGGTTTG	GTCCGCCGAGTCCTTACTGA	CAGTGCCTACAGTCTCGAGTCTGCCAGTG	84
TOP1	TGTAGCAAAGATGCCAAGGT	TGTTATCATGCCGGACTTCT	CCTTCTCCTCCTCCAGGACATAAGTGGA	70
VEGFA	AGTGGTCCCAGGCTGCAC	TCCATGAACTTCACCACTTCGT	ATGGCAGAAGGAGGAGGGCAGAATCA	70
ACTB	GAGCGCGGCTACAGCTT	TCCTTAATGTCACGCACGATTT	ACCACCACGGCCGAGCGG	59

Abbreviations: *ERCC1*=excision repair cross-complementing gene 1; *DPD*=dihydropyrimidine dehydrogenase; *TOP1*=topoisomerase I; *VEGFA*=vascular endothelial growth factor-A. *ACTB*=beta-actin

Supplemental Table 2: Immunostaining results for ERCC1 and DPD

Molecular features		Total N	Prior oxaliplatin-based chemotherapy No	Prior oxaliplatin-based chemotherapy Yes	*P* value
ERCC1					0.44
	negative	146 (45%)	72 (43%)	74 (47%)	
	positive	191 (55%)	97 (57%)	84 (53%)	
DPD					0.027
	negative	202 (61%)	113 (66%)	89 (55%)	
	positive	131 (39%)	57 (34%)	74 (45%)	

(%) indicates the proportion of cases with a specific molecular feature among each group (prior chemotherapy Yes or No)

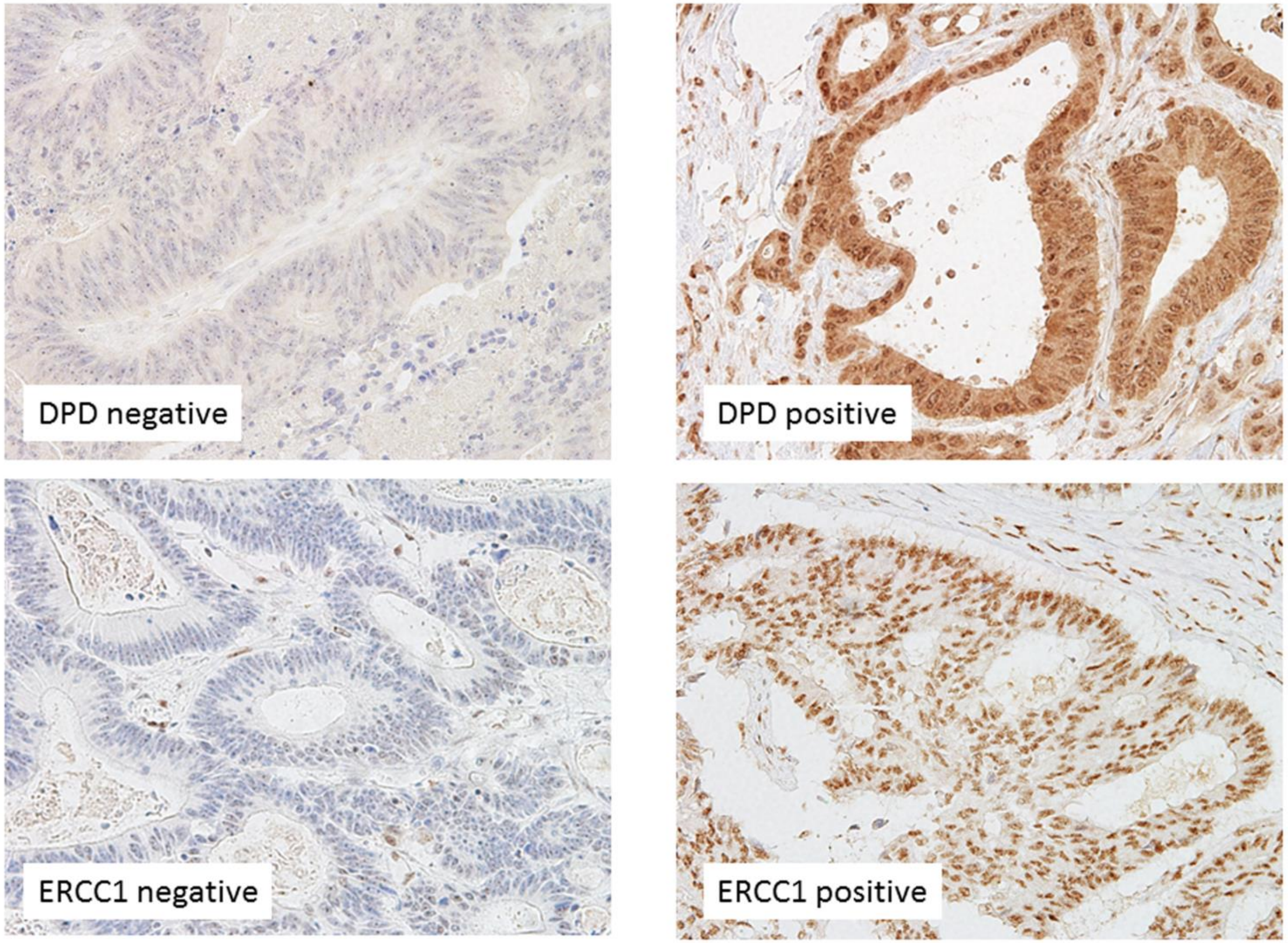

Supplemental Figure

Laboratory Methods

Ten micrometer thick sections were obtained from identified areas with the highest tumor concentration and were then mounted on uncoated glass slides. For histology diagnosis, representative sections were stained with H&E by the standard method. Before microdissection, sections were deparafinized in xylene for 10 min and hydrated with 100%, 95% and finally 70% ethanol solutions. Sections were then washed in H_2O for 30 sec, stained with nuclear fast red (NFR, American MasterTech Scientific, Lodi, CA) for 20 sec and rinsed again in H_2O for 30 sec. Finally, samples were dehydrated with 70% ethanol, 95% ethanol and 100% ethanol solutions for 30 sec each, followed by xylene again for 10 min. The slides were then completely air-dried. The sections of interest were selectively isolated by laser capture microdissection (P.A.L.M. Microsystem, Leica, Wetzlar, Germany), according to the standard procedure.

Blinded tissue samples to be extracted were placed in a 0.5-mL thin-walled tube containing 400 µl of 4 M dithiothreitol (DTT)- GITC/sarc (4 M guanidinium isothiocyanate, 50 mM Tris-HCl, pH 7.5, 25 mM EDTA) (Invitrogen; No. 15577-018). The samples were homogenized and an additional 60 µl of GITC/sarc solution was added. They were heated at 92°C for 30 min and then transferred to a 2-mL centrifuge tube. Fifty microliters of 2 M sodium acetate was added at pH 4.0, followed by 600 µl of freshly prepared phenol/chloroform/isoamyl alcohol (250:50:1). The tubes were vortexed for 15 sec, placed on ice for 15 min and then centrifuged at 13,000 rpm for 8 min in a chilled (8°C) centrifuge. The upper aqueous phase was carefully removed and placed in a 1.5-mL centrifuge tube. Glycogen (10 µl) and 300–400 µl of isopropanol were added and the samples were vortexed for 10–15 sec. The tubes were chilled at -20°C for 30–45 min to precipitate the RNA. The samples were then centrifuged at 13,000 rpm for 7 min in an 8°C centrifuge. The supernatant was poured off and 500 µl of 75% ethanol was added. The tubes were again centrifuged at 13,000 rpm for 6 min in a chilled (8°C) centrifuge. The supernatant

was then carefully poured off, so as not to disturb the RNA pellet, and the samples were quick-spun for another 15 sec at 13,000 rpm. The remaining ethanol was removed and the samples were left to air-dry for 15 min. The pellet was resuspended in 50 µl of 5 mM Tris. And finally, cDNA was prepared as based on the method by Lord and colleagues.

Quantification of 4-interest-genes and an internal reference gene (beta-actin) was done using a fluorescence based real-time detection method (ABI PRISM 7900 Sequence detection System, TaqMan®, Perkin-Elmer (PE) Applied Biosystem, Foster City, CA). The PCR reaction mixture consisted of 1,200 nM of each primer, 200 nM of probe, 0.4 U of AmpliTaq gold polymerase, 200 nM each of dATP, dCTP, dGTP and dTTP, 3.5 mM of $MgCl_2$ and 1xTaqman buffer A containing a reference dye. The final volume of the reaction mixture was 20 µl (all reagents from PE Applied Biosystems, Foster City, CA). Cycling conditions were 50°C for 2 min, 95°C for 10 min, followed by 46 cycles of 95°C for 15 sec and 60°C for 1 min. The primers and probes used are listed in Supplemental Table I. TaqMan® measurements yield Ct values that are inversely proportional to the amount of cDNA in the tube. For example, a higher Ct value means that more PCR cycles are required to reach a certain level of cDNA detection. Gene expression values (relative mRNA levels) are expressed as ratios (differences between the Ct values) between the gene of interest and an internal reference gene (beta-actin). This reference gene provides a baseline measurement for the amount of RNA isolated from a specimen.
